# The complete mitochondrial genome of *Euonymus alatus* (celastraceae, *Euonymus* L.)

**DOI:** 10.1080/23802359.2020.1860702

**Published:** 2021-01-16

**Authors:** Ting-Ting Zhang, Jing Sun, Liang Xu, Yan-Yun Yang, Zhi-Lai Zhan, Yan-Ping Xing, Rong Zhao, Sheng-Nan Li, Da-Chuan Zhang, Ting-Guo Kang

**Affiliations:** a School of Pharmacy, Liaoning University of Traditional Chinese Medicine, Dalian, China; b Traditional Chinese Medicine Resource Center, Chinese Academy of Traditional Chinese Medicine, Beijing, China

**Keywords:** Complete mitochondrial genome, phylogenetic tree, *Euonymus alatus*

## Abstract

The complete mitochondrial genome of medicinal plant, *Euonymus alatus*, was sequenced for the first time. The genome sequence is 1,045,106 bp in length (GenBank accession number MW009108), with 44.98% GC contents. There are 72 genes in the genome, including 41 known protein-coding genes (PCGs), 22 transfer RNAs (tRNAs), and three ribosomal RNAs (rRNAs). The phylogenetic trees of 28 species are constructed using the maximum-likelihood method. The information will provide references for phylogenetic research.

*Euonymus alatus* is an important medicinal plant of *Euonymus* L., which is distributed over China, Japan and Korea (Qin et al. 2011). Its branches with thrombus wings are the medicinal parts and are commonly called ‘Guijianyu’ in China (National Pharmacopoeia Commission 1963). Many chemical constituents have been isolated and identified from *E. alatus*, including sesquiterpenoids, diterpenoids, triterpenoids, flavonoids, phenylpropanoids, lignans, steroids, alkaloids and other compounds (Zhou et al. 2009; Zhai et al. 2016). Some studies have shown that the extracts and compounds from *E. alatus* have a wide pharmacological effects, including anti-diabetes, anti-inflammatory, anti-tumor, liver protection and so on (Fan et al. 2020). At present, there are reports on molecular studies of Celastraceae, but the related research of *E. alatus* is quite limited (Simmons et al. 2001).

Fresh leaves of *E. alatus* were collected from Dandong, China (E 124°39′79.85″, N 40°15′08.55″). Professor Tingguo Kang of Liaoning University of Traditional Chinese Medicine identified the certificate specimen (*E. alatus* number: 10162200522008LY). The plant samples were deposited in the herbarium of Liaoning University of Traditional Chinese Medicine, and the genomic DNA was stored in the Key Laboratory of Traditional Chinese Medicine. Total genomic DNA was extracted with a Plant Tissue Mitochondrial DNA Extraction Kit (Genmed Scientific Inc., Arlington, MA, USA). One short sequencing library (*ca*. 300 bp) were constructed and sequenced using Illumina Novaseq 6000 platform. Genome assembly was performed by SOAP denovo 2.04 (Luo et al. 2012). The mitochondrial genes were annotated using the online DOGMA tool (http://dogma.ccbb.utexas.edu/index.html) (Wyman et al. 2004).

The assembly of the *E. alatus* resulted in a final sequence of 1,045,106 bp in length (GenBank accession number MW009108), and GC content was 44.98%. The structure of mitochondrial genome was a typical circle type. There were 72 genes annotated, including 41 known protein-coding genes, 22 tRNAs, and three rRNAs. The total length of protein-coding genes was 33,960 bp, accounting for 3.25% of the total genome length. The average length of tRNA was 75 bp, while the rRNA was 1828 bp. In addition, we found that 10 genes (*nad2*, *nad7*, *nad5*, *nad1*, *nad4*, *cox2*, *ccmC*, *ccmFc*, *rps3*, *rps10*) contained 24 introns.

Phylogenetic trees are widely used in genetic and evolutionary studies of various organisms. Using the maximum-likelihood method, we constructed a phylogenetic tree based on the complete mitochondrial genome, including 27 angiosperms (including *E*. *alatus*, WM) and one species (*Taxus cuspidata*) of outgroup. We also listed all species accession numbers used in this study. The maximum-likelihood tree showed the position of *E. alatus* in all species, and *E. alatus* can be clustered into a single branch. Additionally, *T. cuspidata* was far from the other species (Figure 1). The phylogenetic tree constructed based on the mitochondrial genome sequence of *E. alatus* will help its classification and evolutionary research.

**Figure 1.**
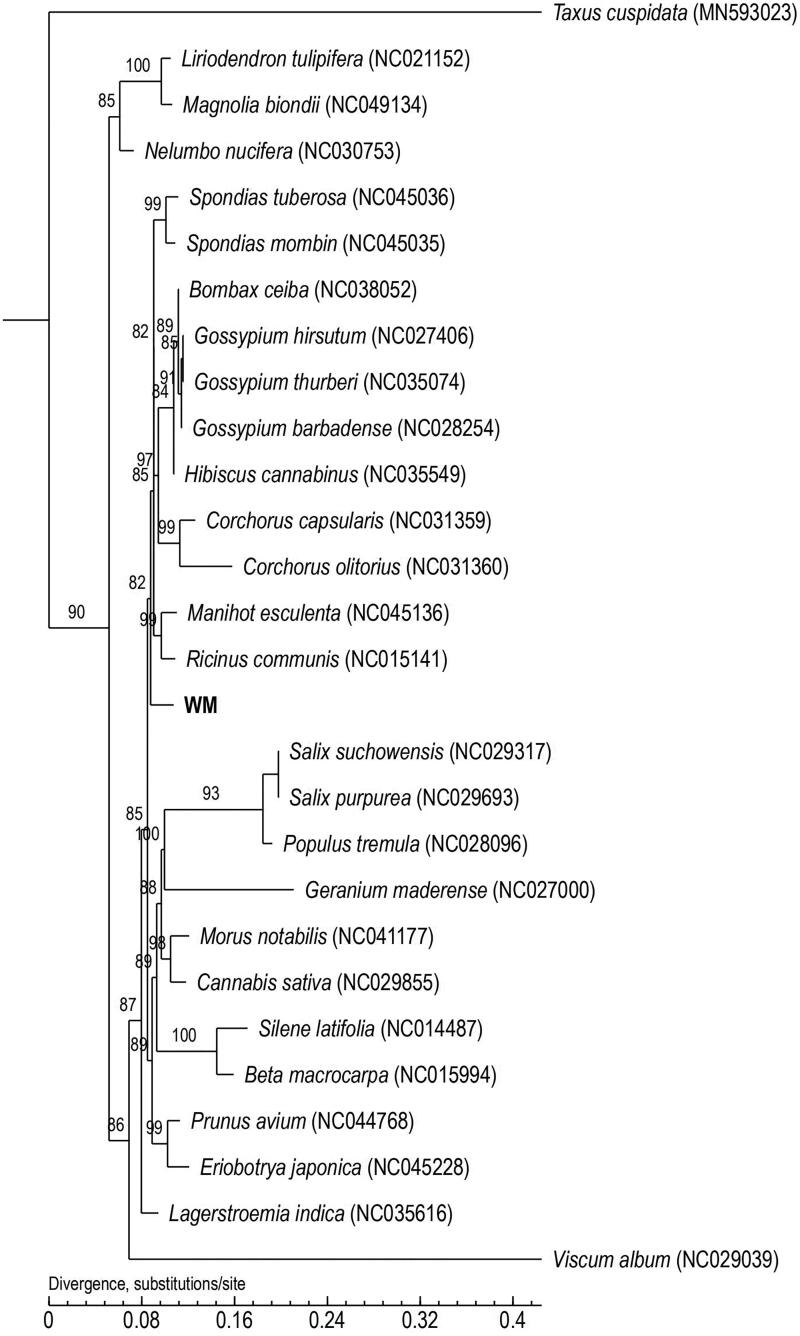
Maximum-likelihood (ML) phylogenetic tree of *E*. *alatus* (WM) and 27 other species. Number above each node indicates the ML bootstrap support values. The model GTR + I + G was selected for the ML analyses with 1000 bootstrap replicates to calculate the bootstrap values.

## Data Availability

The data that support the findings of this study are openly available in NCBI at https://www.ncbi.nlm.nih.gov/search/all/?term=MW009108, reference number MW009108.
